# Case Report: Fulminant meningococcus-meningitis under treatment with ravulizumab despite complete vaccination—a rare but severe complication

**DOI:** 10.3389/fmed.2026.1943514

**Published:** 2026-09-23

**Authors:** Gábor Kozák, Stefan Förster, Michael Haap, Reimer Riessen, Samuel Böhm-Gonzalez, Jan-Hendrik Stahl, Alexander Grimm, Ulf Ziemann, Siri Göpel, Josua Kegele, Annerose Mengel, Katharina Feil

**Affiliations:** 1 Department for Vascular Neurology, Centre for Neurology, University of Tübingen, Tübingen, Germany; 2 Hertie-Institute for Clinical Brain Research, University of Tübingen, Tübingen, Germany; 3 Department of Medicine, Medical Intensive Care Unit, University Hospital Tübingen, Tübingen, Germany; 4 Department for Epileptology, Centre for Neurology, University of Tübingen, Tübingen, Germany; 5 Division of Infectious Diseases, Department of Internal Medicine I, University Hospital Tübingen, Tübingen, Germany

**Keywords:** complement therapies, meningitis, meningitis vaccines, myasthenia gravis, ravulizumab

## Abstract

**Objectives:**

Complement C5 inhibitors such as ravulizumab are increasingly used for acetylcholine receptor–positive generalized myasthenia gravis (MG) but confer a relevant risk of invasive meningococcal infection. We report a rare case of fulminant meningococcal meningitis despite complete vaccination, highlighting the limits of current preventive strategies.

**Methods:**

Case report

**Results:**

A 41-year-old woman with acetylcholine receptor–positive MG was treated with ravulizumab after two-dose series meningococcal vaccination (MenACWY and MenB). One month after therapy initiation, she presented with nausea, vomiting and rapidly developed impaired consciousness. CSF showed neutrophilic pleocytosis, hypoglycorrhachia, and high lactate, consistent with bacterial meningitis. Rapid testing and culture confirmed *Neisseria meningitidis serotype Y*. Despite immediate broad-spectrum antibiotics, neurological deterioration required intubation and intensive care. The patient recovered after 18 days of intravenous antibiotics and was switched from ravulizumab to rozanolixizumab.

**Discussion:**

This case illustrates that vaccination does not abolish the risk of fulminant meningococcal infection under complement inhibition. Awareness, patient information, and rapid initiation of antibiotics are critical. Clinicians should counsel patients and relatives that meningitis may occur despite vaccination and promote early recognition strategies.

## Introduction

Ravulizumab, a long-acting complement C5 inhibitor, was recently approved—among other indications (1, 2), for the treatment of acetylcholine receptor (AChR)-positive MG as an add-on therapy (3). By blocking the formation of the membrane attack complex, it effectively reduces neuromuscular junction damage but also impairs innate immunity against *Neisseria meningitidis*. Invasive meningococcal disease is therefore a well-known risk, and current guidelines mandate vaccination prior to treatment. However, vaccination decreases but does not eliminate risk. We present the case of a patient with MG who developed life-threatening meningococcal meningitis requiring intensive care within weeks of initiating ravulizumab.

## Case description

The 41-year-old woman was diagnosed with acetylcholine receptor-positive MG (MGFA class II) in 2015. The initial treatment included the surgical resection of a B2-thymoma (R1-resection) with an adjuvant radiotherapy, an immunomodulatory treatment with azathioprine and low dose prednisolone, and a symptomatic treatment with pyridostigmine. Due to progressive symptoms over 5 years from 2019 onwards, complement inhibition with ravulizumab was initiated 6 weeks after completion of the meningococcal vaccination schedule recommended in Germany at the time with MenACWY (Menveo®) and MenB (Bexsero®) vaccines (two doses each). The patient had no previous history of meningococcal vaccination. At the time of the decision regarding therapy change, the Besinger score was 8.

One month later, she presented to the emergency department with nausea and vomiting. Within hours, she developed somnolence and confusion. Lumbar puncture showed cerebrospinal fluid (CSF) with 2,866 leukocytes/µL (predominantly polymorphonuclear cells), glucose 6 mg/dL (serum/CSF ratio 0.06), protein 3,127 mg/dL, and lactate 10.4 mmol/L.

Intravenous antibiotic therapy with ceftriaxone (2 g twice daily) and ampicillin (2 g six times daily) was initiated immediately in the emergency department. Following recognition of the patient's immunosuppressed status, the antibiotic regimen was subsequently changed to meropenem (2 g three times daily) and vancomycin (1 g twice daily) (4). The rapid diagnostic test of meningitis confirmed a penicillin sensitive Neisseria meningitis. Upon further deterioration of neurological status, intubation became necessary, and the patient was referred to the neurological intensive care unit (ICU). A follow-up CT showed a diffuse brain edema, but no decompressive craniectomy was needed. Following the isolation of *Neisseria meningitidis* (serogroup Y) from CSF, antimicrobial therapy was de-escalated to ceftriaxone (2 g twice daily) in accordance with current German guidelines (4).

The patient responded well to the antibiotics, her consciousness improved, allowing for extubation six days after symptom onset. CSF follow-up after 18 days showed 17 cells/µL and lactate of 2.4 mmol/L with normal glucose and protein levels. Antibiotic therapy was ended after 18 days. Prophylactic oral antibiotic therapy with azithromycin 500 mg/day was maintained for 8 weeks after the last ravulizumab administration. Additionally, household members were prescribed prophylactic antibiotics to mitigate the risk of environmental contamination and subsequent patient exposure. Ravulizumab therapy was discontinued, and treatment was switched to the human IgG4 neonatal Fc receptor inhibitor rozanolixizumab. In the following weeks further examinations revealed a thymoma metastasis in the pleura, which could be successfully surgically removed.

## Discussion

Impairments to terminal complement activation increase the risk of invasive meningococcal disease by 7,000–10,000-fold, as they prevent terminal complement-mediated bacterial lysis.

Ravulizumab inhibits complement C5 and thus the assembly of the pore-forming membrane attack complex (MAC) (5). The MAC plays a crucial role not only in the pathomechanism of MG through the destruction of the neuromuscular junction (6), but also physiologically in the elimination of the Gram-negative bacteria, such as *Neisseria meningitidis* by forming pores in its cell wall (7).

The increased susceptibility for bacterial meningitis is a well-known and physiologically understood side effect of such inhibitors (8), and vaccination (or prophylactic antibiotics in case of urgency) is therefore mandatory before initiating a therapy.

There are important differences in guideline recommendations between countries and across different indications. The current U.S. guidelines recommend a three-dose series of 4CMenB vaccination for high-risk patients (at 0, 1–2, and 6 months) (9), compared with the two-dose vaccination schedule recommended in Germany and Europe (10). In contrast, the two-dose series vaccination regimen for MenACWY is similar, which is particularly relevant in this case given that the infection was caused by *N. meningitidis* serogroup Y. In Germany, serogroup B was the most prevalent until the COVID-19 pandemic, accounting for approximately 80% of cases. In contrast, serogroup Y, which was identified in this patient, has increased markedly in frequency during the post-pandemic years, such that in 2025, serogroups B and Y accounted for 45.2% (*n* = 123) and 41.2% (*n* = 114) of cases, respectively based on data from the Robert Koch Institute (RKI) (11). For other indications, such as atypical hemolytic uremic syndrome, recommendations are more permissive regarding prophylactic antibiotic treatment (12). In patients with gMG, however, because antibiotics may exacerbate symptoms of the underlying disease, antibiotic prophylaxis is recommended only in cases of incomplete vaccination (13). As discussed above, the definition of complete vaccination varies slightly between countries.

However, these preventive measures do not completely prevent infection; they only reduce its risk (14, 15). Further, the role of serological monitoring of the vaccine response in clinical practice is currently unclear due to limited data. An evaluation of meningococcal vaccination titers in 324 patients in the UK who were treated with complement inhibitors for paroxysmal nocturnal hemoglobinuria (PNH) showed that breakthrough infections are possible even with additional antibiotic prophylaxis with penicillins and titer levels considered protective (16). The evaluation demonstrated that a serological response to vaccination could be attained following a single revaccination in cases where the initial attempt had proved unsuccessful.

Our current case highlights two unusual and clinically very important aspects: First, infection occurred despite complete immunization with MenACWY and MenB vaccines. Possible explanations include limited serotype coverage, variable immune response, and incomplete protection at the mucosal level. Importantly, patients receiving ravulizumab treatment are not therapy-naïve, as it is only approved as an add-on therapy. This means that vaccination will mostly take place under immunosuppression, as in our patient. Although there is currently no evidence that prior immunosuppression with agents such as azathioprine increases the risk of ravulizumab-related meningitis, it is known that the efficacy of vaccination is reduced during ongoing immunosuppressive treatment (17). Additionally, the patient might have been more susceptible to infections as concomitant tumor disease, i.e., metastasis of thymoma Matsuoka 2B, was found during recovering.

Second, the clinical course was fulminant, requiring intensive care, underscoring that preventive strategies reduce, but do not abolish this risk. Therefore, it is important to inform the patients and close relatives about the early clinical symptoms, as immediate antibiotic treatment is decisive for the outcome in bacterial meningitis (18). One recent expert consensus paper on therapy with complement inhibitors even suggested that patients should be equipped with single-shot antibiotics, e.g., ciprofloxacin as “stand-by medication” that can be immediately self-administered in case of meningitis-like symptoms (13). Further, sufficient re-vaccination for ACWY and B is highly recommended every 2–3 years.

Furthermore, environmental and occupational factors should be considered individually. The patient worked as a dental assistant, an occupation involving frequent close contact with patients and potential exposure to respiratory and oral secretions. Although this occupation is not generally considered to confer a high risk of meningococcal infection, frequent close contact with patients may represent a potential source of exposure, particularly in the setting of complement C5 inhibition.

Another relevant clinical consideration is whether complement inhibition must be permanently discontinued after an invasive infection. Current guidelines do not mandate discontinuation, but patient adherence, trust, and risk tolerance must guide shared decision-making. In our case, ravulizumab was discontinued and therapy was switched to rozanolixizumab, a subcutaneously administered Fc receptor inhibitor that does not interfere with the complement system and therefore carries no increased risk of *Neisseria* meningitis.

The present case therefore raises three practical management issues related to the use of complement inhibitors:

- Vaccination against *Neisseria meningitidis* alone might be insufficient, particularly in cases of long-lasting immunosuppression or concomitant disorders, such as tumors. Expert consensus now recommends additional strategies such as prophylactic antibiotics or standby oral antibiotics (e.g., ciprofloxacin) for immediate use if meningitis-like symptoms occur.
- **Patient and caregiver information is crucial.** Awareness of early warning symptoms — headache, fever, neck stiffness or altered consciousness — is decisive for rapid presentation and initiation of antibiotics.
- **Continuation of complement inhibition after meningitis remains debated.** Guidelines do not mandate discontinuation, but treatment adherence, patient trust, and perceived safety must be considered. In this case, ravulizumab was permanently discontinued.

## Conclusion

This case illustrates that fulminant meningococcal meningitis may occur under ravulizumab therapy despite full vaccination. Neurologists prescribing complement inhibitors should counsel patients and caregivers on residual risk, provide clear instructions for early medical consultation, and consider prophylactic or standby antibiotics.

## Data Availability

The original contributions presented in the study are included in the article/Supplementary Material, further inquiries can be directed to the corresponding author.
